# The associations between the Geriatric Nutritional Risk Index and all-cause, cancer-specific, and cardiovascular mortality in the U.S. population: a large-scale pooled survey

**DOI:** 10.1186/s12986-024-00827-7

**Published:** 2024-07-12

**Authors:** Kun Han, Tianhong Wang, Congcong Zou, Tao Li, Leng Zhou

**Affiliations:** 1 Department of Anesthesiology, West China Second University Hospital, Sichuan University, Chengdu, 610041 China; 2 Key Laboratory of Birth Defects and Related Diseases of Women and Children, Sichuan University, Chengdu, 610041 China; 3 Department of Anesthesiology, West China Hospital, Sichuan University, Chengdu, 610041 China; 4 Department of Anesthesiology, Laboratory of Mitochondria and Metabolism, West China Hospital, National Clinical Research Center for Geriatrics, Sichuan University, Chengdu, 610041 Sichuan China

**Keywords:** Geriatric nutrition risk index, Mortality, National health and Nutrition examination survey

## Abstract

**Background:**

Previous studies have reported a close association between the Geriatric Nutritional Risk Index (GNRI) and various conditions. However, the association between the GNRI and mortality remains unclear. To examine the correlation between the GNRI and all-cause, cancer-specific, and cardiovascular mortality, this study was performed.

**Methods:**

We analyzed elderly participants in the National Health and Nutrition Examination Survey from 2005 to 2016. The GNRI was calculated using body mass index and serum albumin. Kaplan-Meier survival curves were drawn to compare the survival probability between the normal and decreased GNRI groups. Weighted multivariate Cox regression and restricted cubic spline (RCS) models were employed to determine the linear and non-linear associations of the GNRI with all-cause, cancer-specific, and cardiovascular mortality.

**Results:**

A total of 3,276 participants were included in the analysis. The Kaplan-Meier survival curve showed that the decreased GNRI group had a lower survival probability for all-cause mortality and cancer-specific mortality (*P* < 0.001) but not for cardiovascular mortality (*P* > 0.05). In the full regression models, the decreased group had a higher risk of all-cause mortality (HR = 1.67, 95% CI = 1.21–2.30, *P* = 0.002), and cancer-specific mortality (HR = 2.20, 95% CI = 1.32–3.67, *P* = 0.003) than the normal group. For cardiovascular mortality, no significant association with GNRI (HR = 1.39, 95% CI = 0.60–3.22, *P* = 0.436) was detected. Notably, the RCS analysis identified a linear downward trend between the GNRI and all-cause, alongside cancer-specific mortalities (all *P* for overall < 0.05). The time-dependent Receiver Operating Characteristic (ROC) analysis unveiled the predictive power of the GNRI for 5-year all-cause mortality, cancer mortality, and cardiovascular mortality was 0.754, 0.757, and 0.836, respectively, after adjusting for covariates.

**Conclusions:**

Individuals with a decreased GNRI had increased risks of all-cause, and cancer-specific mortality. There were linear associations of the GNRI with all-cause, and cancer-specific mortality. Nutritional status should be carefully monitored, which may improve the overall prognosis for the general population.

**Supplementary Information:**

The online version contains supplementary material available at 10.1186/s12986-024-00827-7.

## Background

Malnutrition among older adults is a significant health issue associated with increased rates of diverse conditions and even mortality [1]. It also leads to physical deterioration, severely impacting daily activities and overall quality of life. The elderly population is susceptible to physical functional impairment and various diseases, which may include gastrointestinal structural changes, reductions in gastrointestinal hormone secretion, reduced activity and appetite, comorbidities, isolation, mental depression, and economic problems. Consequently, elderly individuals often experience insufficient nutrient intake [1]. Thus, it is crucial to pay more attention to the nutritional needs of the elderly population.

The Geriatric Nutritional Risk Index (GNRI), which is calculated using body mass index (BMI) and serum albumin, has been developed to define malnutrition [2]. The GNRI is a straightforward dietary index that has a strong association with the prognosis of various diseases including diabetes, heart failure, and cancer [3–5]. Several studies on patients with malignant tumors have shown that the GNRI is more advantageous than serum albumin levels, body weight, or BMI alone for prognostic evaluation [6, 7]. Patients with non-ST-segment elevation acute coronary syndrome who have a lower GNRI experience a worse prognosis when undergoing percutaneous coronary intervention [8]. These studies indicate that GNRI may be a useful predictive index for poor prognosis and mortality. Three previous studies found that GNRI were closely associated cardiovascular or all-cause mortality in individuals with diabetes, chronic obstructive pulmonary disease, or hypertension [9–11]. However, these conditions are correlated with malnutrition, possibly justifying the association of GNRI with mortality [12, 13]. Thus, assessing the association of GNRI with mortality in the general population can yield more solid conclusions, reducing bias from the above-mentioned comorbidities. In addition, Yu et al. investigated the association of GNRI with mortality in the elderly Chinese [1]. Their study only focuses on all-cause mortality, which could not provide information on cause-specific mortality. Therefore, further studies targeting cause-specific mortality in the general population are requisite.

The National Health and Nutrition Examination Survey (NHANES) is a comprehensive cross-sectional survey that gathers data on the health and nutrition of the U.S. population [14]. With wide sample coverage, the NHANES database offers extensive information on demographics, socioeconomics, diet and health, physiological measurements, laboratory tests, and more throughout the U.S. [14]. Therefore, this study aimed to investigate the correlation between malnutrition and mortality among the elderly population in the U.S. by utilizing data from the NHANES.

## Materials and methods

### Study design and sample

NHANES, conducted by the US Center for Disease Control and Prevention, is a cross-sectional survey employing a multistage probability sampling technique to ensure a representative sample of the non-institutionalized population in the U.S. The survey employs various data collection methods, such as face-to-face or phone interviews, questionnaires, laboratory testing, and physical examinations. Home interviews are conducted with participants, while mobile examination centers are utilized for physical examinations and blood sample collection. The study received approval from the National Center for Health Statistics Institutional Ethics Review Board, with all participants providing written consent. NHANES has been conducting annual surveys since 1999, with data released to the public every two years.

**Fig. 1:**
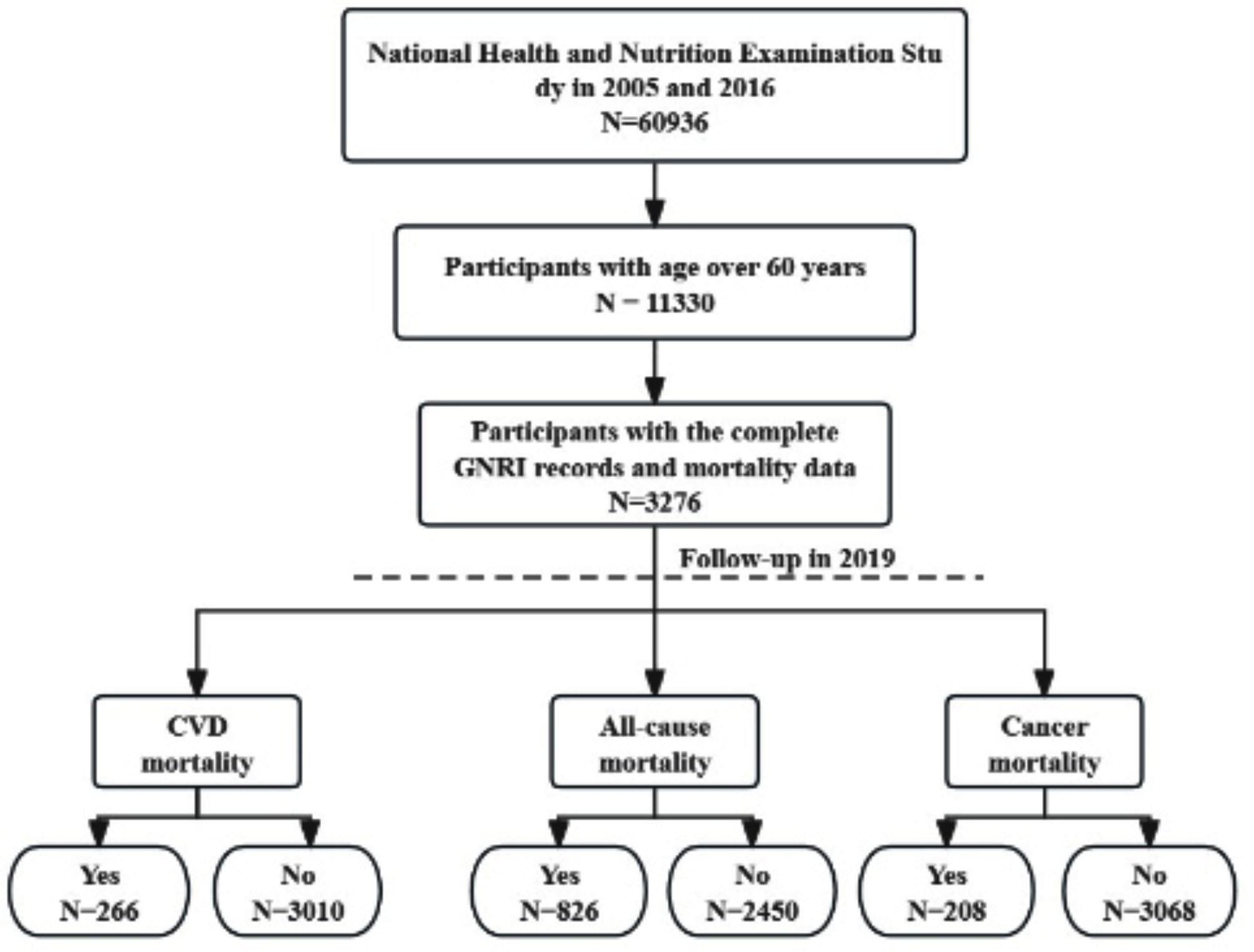
The flow chart

Leveraging the open database, we collected data from six survey cycles spanning the years 2005 to 2016. The six cycles included a total of 60,936 participants. Individuals under the age of 60 were not considered in this study. NHANES has gathered comprehensive data on sociodemographic factors, lifestyle aspects (including sleep duration), medical history, and health-related information, which incorporates clinical measurements such as blood pressure, fasting blood glucose levels, and serum lipids (including triglycerides and high-density lipoprotein cholesterol), as well as self-reported use of medications for various health conditions. Participants without missing values for any covariates, GNRI and mortality records were included in the final analysis (Fig. 1). The final sample size for the cross-sectional examination comprised 3,276 participants.

### Exposure measures: geriatric nutritional risk index

The GNRI was computed using the following formula: GNRI = [1.489 × serum albumin (g/L)] + (41.7 × weight (kg) / ideal weight (kg)). The ideal weight was determined through the Lorenz equation: 22 × square of height. When the patient’s weight surpassed the ideal weight, the weight to ideal weight ratio was fixed at 1 [2]. Patient classification was established based on specific thresholds: a GNRI value of less than 98 indicated a potential risk, and a value exceeding 98 indicated no risk [9].

### Outcome: all-cause and cause-specific mortality

The determination of all-cause mortality was based on National Death Index (NDI) records. Cause-specific mortality was identified using the International Classification of Diseases, 10th Revision (ICD-10). Cardiovascular disease (CVD) death was defined by the ICD-10 codes I00-I09, I11, I13, and I20-I51, while cancer-specific mortality was determined by the ICD-10 code range C00-C97 [15].

### Covariate assessments

According to prior studies, we included age, sex, sleep duration, family poverty to ratio, physical activity, smoking status, alcohol use, cardiovascular disease, and diabetes. Age was divided into 60–80 years and over 80 years. Participants provided self-reported information on their typical weekday or workday sleep duration. From 2005 to 2014, sleep duration was derived from a question posed to NHANES participants regarding their average nightly hours of sleep: “How much sleep do you get (hours)?” During the 2015 and 2018 cycles, sleep duration was determined based on the following query: “How much sleep do you usually get at night on weekdays or workdays?” Sleep duration was recorded as hours/day. The family poverty ratio was grouped as 0-1.4, 1.5–3.4, and ≥ 3.5. Smoking status was defined as former, never, or present. Alcohol use was defined as former, never, mild, moderate, or heavy [14]. In this study, heavy alcohol use was defined as consuming three or more drinks per day for females or four or more drinks per day for males, or engaging in binge drinking on five or more days per month. Moderate alcohol use was defined as consuming two or more drinks per day for females or three or more drinks per day for males or engaging in binge drinking on two or more days per month. Any alcohol consumption below these thresholds was considered mild alcohol use. The level of physical activity was assessed using metabolic equivalents per week [16]. Diabetes mellitus was defined as a self-reported diagnosis of diabetes, a glycohemoglobin HbA1c level greater than 6.5%, a fasting glucose level equal to or greater than 7.0 mmol/L, a random blood glucose level equal to or higher than 11.1 mmol/L, a 2-hour oral glucose tolerance test blood glucose level equal to or higher than 11.1 mmol/L, or the use of diabetes medication or insulin [17].

### Statistical analysis

The demographic characteristics of the participants in the decreased GNRI group, normal GNRI group, and overall group were described. Continuous variables were shown as the mean and standard deviation, and categorical variables were presented as numbers and proportions. Cumulative mortality rates were estimated by the Kaplan-Meier method. The Log-rank test was used to test the difference between the GNRI groups. Weighted multivariate Cox regression models were employed to determine the association of the GNRI group with all-cause, cancer-specific, and cardiovascular mortalities using wtmec2 year weight. After adjusting for covariates, “log-log” plots were drawn to assess the proportional-hazards assumption. Three models were fitted in turn. In detail, model one was a crude model without covariates. Model two was adjusted for age and sex. Model three was adjusted for model two plus educational levels, family poverty ratio, sleep duration, physical activity, smoking status, alcohol consumption, CVD and diabetes. The effects were estimated using hazard ratios (HRs) and 95% confidence interval (95% CI). Because the GNRI did not meet the proportional hazards assumption in the analysis related to cardiovascular mortality, we included an interaction term between follow-up time and GNRI in the model to adjust for the effect of time. The weighted restricted cubic spline using three knots placed in the 25%, 50% and 75% percentiles was modeled to fit the nonlinear associations of the GNRI with all-cause mortality, cancer-specific mortality, and cardiovascular mortality. The time-dependent Receiver Operating Characteristic (ROC) analysis was used to assess the predictive power of GNRI for the 5-year mortality risk.

All the statistical analyses were performed using STATA software version 18.0 (Stata Corporation, College Station, TX, U.S.). *P* < 0.05 was considered to indicate statistical significance.

## Results

### Characteristics of the included participants

From 2005 to 2016, a total of 3276 participants were included in the analysis at baseline (Table 1). The median follow-up time was 8 years. There were 826 cases attributed to all-cause mortality, 266 cases attributed to cardiovascular mortality, and 208 cases attributed to cancer mortality (Fig. 1). Of those, the GNRI decrease group and GNRI normal group enrolled 232 (7.1%) and 3044 (92.9%) individuals, respectively. In the GNRI normal group, 2639 participants (86.7%) were younger than 80 years, and 1556 (51.1%) participants were females. In the GNRI decrease group, 194 (83.6%) participants were younger than 80 years, and 128 (55.2%) participants were females. In relative to the GNRI normal group, the GNRI decrease group tended to have lower family poverty ratio (*P* < 0.001) and prevalence of diabetes (*P* = 0.005), and higher cigarette consumption (*P* < 0.001). Notably, the GNRI decrease group was less likely to receive education (*P* = 0.060) and have cardiovascular diseases (*P* = 0.085). Other information is presented in Table 1.

**Table 1:** The characteristics of participants at baseline

Variables	Geriatric Nutritional Risk Index (GNRI)			
	Normal	Decreased	Total	*P* value
N	3,044 (92.9%)	232 (7.1%)	3,276 (100.0%)	
Age (years)				0.187
<80	2,639 (86.7%)	194 (83.6%)	2,833 (86.5%)	
≥80	405 (13.3%)	38 (16.4%)	443 (13.5%)	
Sex				0.065
Female	1,556 (51.1%)	104 (44.8%)	1,660 (50.7%)	
Male	1,488 (48.9%)	128 (55.2%)	1,616 (49.3%)	
Family poverty ratio				< 0.001
0-1.5	1,170 (38.4%)	117 (50.4%)	1,287 (39.3%)	
1.5–3.5	1,101 (36.2%)	82 (35.3%)	1,183 (36.1%)	
≥3.5	773 (25.4%)	33 (14.2%)	806 (24.6%)	
Educational levels				0.060
Below high school	531 (17.4%)	51 (22.0%)	582 (17.8%)	
High school	1,229 (40.4%)	100 (43.1%)	1,329 (40.6%)	
College or over	1,284 (42.2%)	81 (34.9%)	1,365 (41.7%)	
Drinking status				0.189
Never	807 (26.5%)	62 (26.7%)	869 (26.5%)	
Now	923 (30.3%)	58 (25.0%)	981 (29.9%)	
Former	1,314 (43.2%)	112 (48.3%)	1,426 (43.5%)	
Smoking status				< 0.001
Never	1,507 (49.5%)	94 (40.5%)	1,601 (48.9%)	
Now	411 (13.5%)	52 (22.4%)	463 (14.1%)	
Former	1,126 (37.0%)	86 (37.1%)	1,212 (37.0%)	
Physical activity (MET/week)				0.454
Q1	1,023 (33.6%)	85 (36.6%)	1,108 (33.8%)	
Q2	999 (32.8%)	78 (33.6%)	1,077 (32.9%)	
Q3	1,022 (33.6%)	69 (29.7%)	1,091 (33.3%)	
Diabetes				0.005
No	1,692 (55.6%)	151 (65.1%)	1,843 (56.3%)	
Yes	1,352 (44.4%)	81 (34.9%)	1,433 (43.7%)	
Cardiovascular diseases				0.085
No	2,372 (77.9%)	192 (82.8%)	2,564 (78.3%)	
Yes	672 (22.1%)	40 (17.2%)	712 (21.7%)	
Sleep (hours/day)	7.042 (1.455)	7.088 (1.808)	7.046 (1.483)	0.649
Weight (kg)	79.20 (18.06)	71.67 (20.10)	78.70 (18.29)	< 0.001
Height (cm)	165.04 (9.81)	165.30 (9.86)	165.06 (9.81)	0.711
BMI (kg/m^2^)	28.99 (5.79)	26.08 (6.74)	28.80 (5.90)	< 0.001
Albumin (µg/ml)	57.48 (325.40)	114.76 (475.71)	61.27 (337.62)	0.016
Total Cholesterol (mg/dL)	195.06 (43.09)	185.18 (46.01)	194.76 (43.21)	0.029
HDL (mg/dL)	54.46 (16.83)	61.85 (20.59)	54.68 (17.00)	< 0.001
LDL (mg/dL)	113.20 (37.01)	107.38 (41.81)	113.02 (37.17)	0.301

Note. LDL: Low-Density Lipoprotein Cholesterol; HDL: High-Density Lipoprotein Cholesterol

Student’s t test for continuous variables and Chi-square test for categorical variables

### Differences in survival between the GNRI normal and decrease groups

**Fig. 2:**
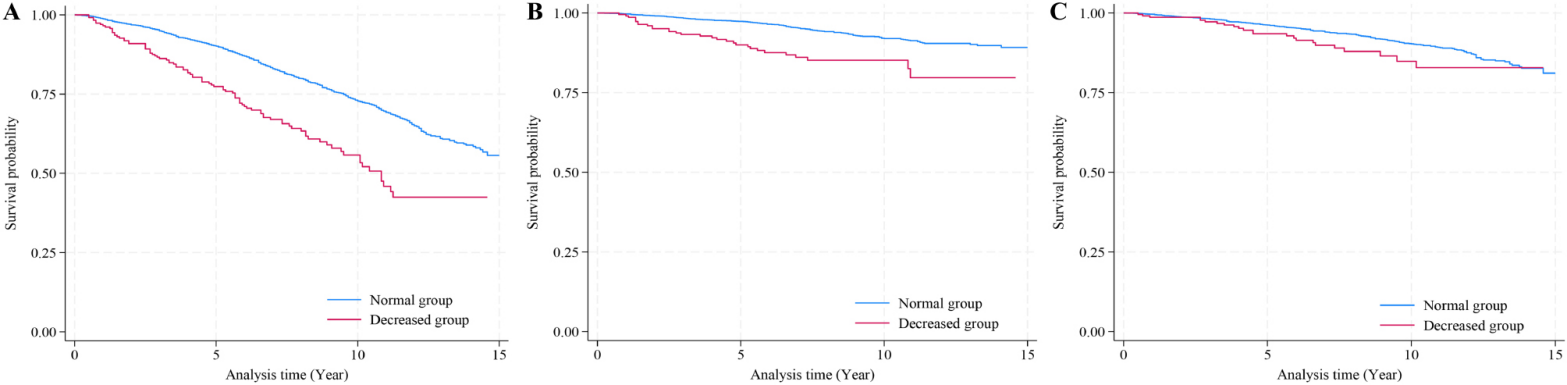
The Kaplan-Meier survival curve by GNRI groups. Plot A indicates all-cause mortality; Plot B indicates cancer-specific mortality; Plot C indicates cardiovascular mortality. The normal and decreased groups are defined by GNRI as revealed in the Methods

As shown in Fig. 2, the Kaplan-Meier survival curve of the GNRI groups showed that the decreased group had a lower survival probability of all-cause mortality and cancer-specific mortality (*P* < 0.001). However, there was no significant difference in cardiovascular mortality between the two groups (*P* > 0.05). As summarized in Table 2, the cumulative mortality rate of the GNRI decrease group was 488.19 (95% CI = 397.97-598.87) per 100,000 for all-cause mortality, 159.19 (95% CI = 111.31-227.68) per 100,000 for cancer-specific mortality, and 122.05 (95% CI = 81.10-183.66) per 100,000 for cardiovascular mortality.

**Table 2:** The cumulative mortality rates of GNRI group across all-cause mortality, cancer mortality, and cardiovascular disease mortality

The cumulative mortality rates (per 100,000)	GNRI groups	
	Normal	Decreased
All-cause mortality	236.19 (236.19, 272.96)	488.19 (397.97, 598.87)
Cancer mortality	61.58 (53.16, 71.32)	159.19 (111.31, 227.68)
Cardiovascular diseases mortality	84.06 (74.13, 95.32)	122.05 (81.10, 183.66)

### Association of the GNRI with all-cause and cause-specific mortality

**Fig. 3:**
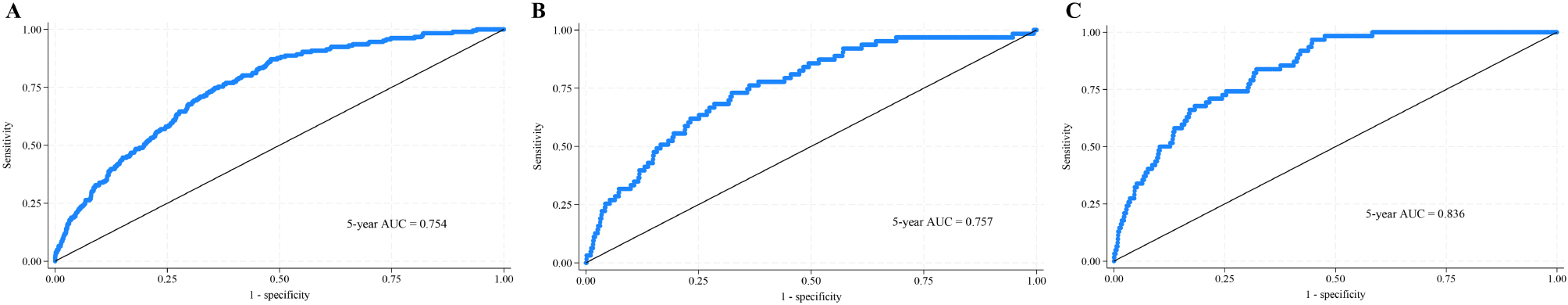
The time-dependent ROC analysis for 5-years mortality risk using GNRI after adjusting for covariates. Plot A, B, C are for all-cause mortality, cancer mortality, and cardiovascular mortality, respectively. All models were adjusted for age, sex, educational levels, family poverty to ratio, sleep duration, physical activity, smoking status, alcohol drinking, cardiovascular diseases and diabetes

Table 3 shows the effects estimation of the GNRI with all-cause and cause-specific mortality. The binary GNRI degree was included in the model as the main exposure, with the normal group as a reference. As shown in Figure S1A-S1B, the proportional-hazards assumption was not violated for all-cause and cancer-specific mortality. However, the proportional-hazards assumption was violated for cardiovascular mortality (Figure S1C). Thus, binary GNRI was included in models for cardiovascular mortality. In the weighted Cox regression model between all-cause mortality and the GNRI, the GNRI decrease group had a higher risk of all-cause mortality, with a HR of 1.67 (95% CI: 1.12–2.30) in the final model. Similarly, we also observed parallel results for cancer-specific and cardiovascular mortality. The GNRI decrease group had a 2.20-fold (95% CI = 1.32–3.67) increase in the risk of cancer-specific mortality, and a 1.39-fold (95% CI = 0.60–3.22) increase in the risk of cardiovascular mortality, though it was not significant. We also observed the similar findings using the continuous GNRI in the model. The HR for GNRI per SD was 0.85 (95% CI = 0.78–0.93, *P* < 0.001) for all-cause mortality, 0.78 (95% CI = 0.67–0.91, *P* = 0.002) for cancer-specific mortality, and 0.95 (95% CI = 0.78–1.16, *P* = 0.611) for cardiovascular mortality. The ROC analysis reported the predictive power of the GNRI for 5-year mortality risk was 0.754, 0.757, and 0.836, respectively, after adjusting for covariates (Fig. 3). In the sensitivity analysis, the associations did not change significantly when adding chronic kidney disease, hypertension, or hyperlipidemia as covariates separately, or when excluding participants who were followed up for less than one year (Table S1 and Table S2).

**Table 3:** Association of GNRI with all-cause mortality, cancer mortality, and cardiovascular diseases mortality using weighted cox regression

Mortality	Model 1		Model 2		Model 3	
	HR (95% CI)	*P* value	HR (95% CI)	*P* value	HR (95% CI)	*P* value
All-cause						
Normal	1	-	1	-	1	-
Decreased	2.06 (1.45–2.92)	< 0.001	1.91 (1.38–2.64)	< 0.001	1.67 (1.21–2.30)	0.002
Continuous per SD	0.78 (0.71–0.85)	< 0.001	0.85 (0.77–0.94)	< 0.001	0.85 (0.78–0.93)	< 0.001
**Cancer**						
Normal	1	-	1	-	1	-
Decreased	2.74 (1.66–4.54)	< 0.001	2.60 (1.58–4.28)	< 0.001	2.20 (1.32–3.67)	0.003
Continuous per SD	0.72 (0.63–0.82)	< 0.001	0.76 (0.66–0.89)	< 0.001	0.78 (0.67–0.91)	0.002
**CVD** ^**a**^						
Normal	1	-	1	-	1	-
Decreased	2.09 (0.92–4.75)	0.077	1.71 (0.78–3.74)	0.178	1.39 (0.60–3.22)	0.436
Continuous per SD	0.84 (0.73–0.97)	0.016	0.98 (0.81–1.18)	0.802	0.95 (0.78–1.16)	0.611

Model 1 was crude model without covariate; Model 2 was adjusted for age and sex; Model 3 was adjusted for model two plus educational levels, family poverty to ratio, sleep duration, physical activity, smoking status, alcohol drinking, cardiovascular diseases and diabetes. SD: Standard deviation. ^**a**^ time-dependent binary GNRI was included in model

### The dose-response association of the GNRI with all-cause and cause-specific mortality

**Fig. 4:**
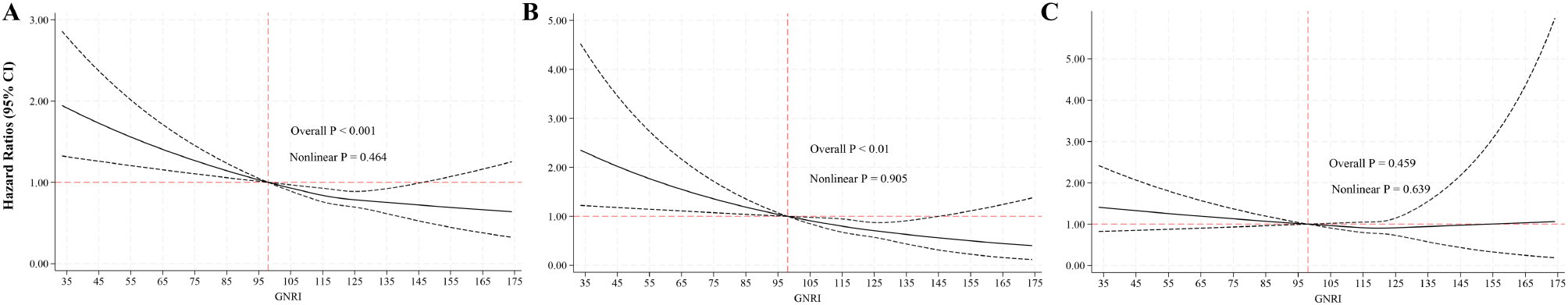
Restrict cubic splines fitting of GNRI with all-cause, cancer, and cardiovascular diseases mortality. Plot A indicates all-cause mortality; Plot B indicates cancer-specific mortality; Plot C indicates cardiovascular mortality

We modeled a restricted cubic spline to smooth the association between an increased GNRI and various kinds of mortality. We did not find any non-linear association between the GNRI and mortality (*P* > 0.05). In contrast, we observed a decreased linear association of GNRI with all-cause and cancer-specific mortality, which indicated negative association of GNRI scores and lifespan loss in elderly individuals (Fig. 4).

## Discussion

This study aimed to investigate the association between GNRI and survival outcomes, using a large sample size. In our study, we analyzed data from 3,276 participants who were part of the NHANES conducted between 2005 and 2016. We found that individuals with a decreased GNRI had increased risks of all-cause and cancer-specific mortality. Notably, there were linear associations of the GNRI with all-cause, and cancer-specific mortality.

GNRI is a widely used index reflecting the overall nutritional status. This index is derived by combining body weight, ideal body weight, and concentration of serum albumin. Previous studies have indicated a correlation between body weight and body cell mass (BCM) [17], which is an indicator of the number of functional cells in the body and plays a pivotal role in substance and energy metabolism [18]. Consequently, a lower BMI poses a threat to normal physiological processes and heightens the likelihood of adverse clinical outcomes. Serum albumin is also a nutritional indicator. It is used to predict the risk of postoperative infection, complications, and mortality [19]. Human serum albumin plays a crucial role in transporting fatty acids, cholochromes, amino acids, steroid hormones, metal ions, and therapeutic molecules in body fluids while maintaining normal blood osmotic pressure. Therefore, a deficiency in serum albumin can impair physiological functions, such as causing osmotic pressure disorders, hindering the delivery of therapeutic factors to inflammatory sites, and preventing steroid hormones from reaching target organs [1].

In this study, malnutrition was found to increase all-cause and cancer-specific mortality, consistent with earlier findings [20–24]. A meta-analysis revealed that a lower GNRI was associated with an increased risk of all-cause mortality in observational studies [25]. A low GNRI in older adults has various detrimental health effects. It can lead to a weakened immune system, impaired wound healing, and muscle weakness. Consequently, these negative outcomes significantly reduce the life expectancy of elderly people and increase the risk of mortality. Notably, an increased but insignificant HR of cardiovascular mortality was identified for the decreased GNRI group. This may be attributed to the phenomenon that individuals with lower GNRI generally have lower low-density lipoprotein and total cholesterol levels, which are widely accepted biomarkers for cardiovascular conditions [26]. Thus, the protective effects of decreased low-density lipoprotein and total cholesterol may offset the harm from malnutrition and lead to insignificance. Additionally, the dose-response associations of GNRI with mortality highlight the continuously increasing harm associated with the severity of malnutrition. Thus, intervention for malnutrition should be timely and thorough.

The relationship between malnutrition and mortality is not driven by a single factor, but rather a combination of multiple mechanisms. Malnutrition can impact T cells by reducing inflammation, cytokine production, and T cell responses, resulting in a state of immunosuppression. Therefore, malnourished patients are at a high risk of experiencing complications related to infection which can ultimately lead to death [27]. Malnutrition affects nearly every organ and system in the human body, resulting in mental and physical decline. Malnutrition not only directly harms the body but also exacerbates underlying diseases, leading to secondary malnutrition and ultimately increasing both mortality and morbidity rates [28]. The high predictive value of the GNRI can be attributed to its incorporation of two important markers of malnutrition: albumin and body weight [2]. Serum albumin levels are a significant indicator of malnutrition, and low levels have been shown to be closely associated with surgical site infections, other complications, and poor survival, regardless of the specific type of cancer. The immunomodulatory role of albumin is widely recognized, as hypoalbuminemia can lead to reduced activation of macrophages and a weaker cell-mediated immune response against cancer cells [29]. By considering the ratio of current body weight to ideal body weight in the calculation, the GNRI also takes into account the patient’s BMI. A low BMI is a well-established marker for poor prognosis in cancer patients [30]. Cardiac dysfunction and congestion have been identified as the primary factors contributing to malnutrition. Hypoalbuminemia has been established as a risk factor for heart failure development [31].

There are several strengths and limitations in the current study. We conducted analyses using a large, nationally representative sample and adjusted for demographic, examination, and laboratory covariates to ensure credible and generalizable associations. However, there are certain limitations that should be acknowledged. First, since the study participants were all from the U.S., caution should be taken when extrapolating the conclusions to other countries and regions. Second, reverse causation is possible since individuals may modify their dietary consumption and nutritional status when they begin to experience symptoms of illness. Third, while we accounted for most confounding factors, we cannot completely dismiss the possibility of residual confounding due to unmeasured or unknown variables. Of note, some covariates, such as sleep duration were evaluated based on self-report instead of objective measurement. The definition of all-cause and cause-specific mortality was based on the codes of ICD-10 rather than more accurate field visits. These limitations may bring bias to the results. Future randomized controlled trials will be necessary to confirm the association between the GNRI and all-cause and cause-specific mortality.

## Conclusions

In conclusion, this study revealed that a decreased GNRI was significantly associated with elevated risks of all-cause and cancer-specific mortality. There were linear associations of GNRI with all-cause and cancer-specific mortality. Nutritional status should be carefully monitored, which may improve the overall prognosis for the general population.

### Electronic supplementary material

Below is the link to the electronic supplementary material.

Supplementary Material 1

Supplementary Material 2

Supplementary Material 3

## Data Availability

No datasets were generated or analysed during the current study.

## Abbreviations

BCM: Body cell mass
BMI: Body mass index
CVD: Cardiovascular disease
GNRI: Geriatric Nutritional Risk Index
HR: Hazard ratio
NHANES: National Health and Nutrition Examination Survey
NDI: National Death Index; RCS: restricted cubic spline
