# Supplementary material for: The associations between the Geriatric Nutritional Risk Index and all-cause, cancer-specific, and cardiovascular mortality in the U.S. population: a large-scale pooled survey
Source: Nutr Metab (Lond). 2024 Jul 12;21:48. doi: 10.1186/s12986-024-00827-7 (PMC11245820; doi:10.1186/s12986-024-00827-7)
Supplement: Supplementary file 1 — Supplementary Material 1 [file 12986_2024_827_MOESM1_ESM.docx]

**Table S1.** The association of GNRI with all-cause mortality, cancer mortality, and cardiovascular diseases mortality after including CKD or hypertension or hyperlipidemia as covariates using weighted cox regression.

| **Mortality** | **CKD** | | **Hypertension** | | **Hyperlipidemia** | |
| --- | --- | --- | --- | --- | --- | --- |
|  | **HR (95% CI)** | ***P* value** | **HR (95% CI)** | ***P* value** | **HR (95% CI)** | ***P* value** |
| **All-cause** |  |  |  |  |  |  |
| Normal | 1 | - | 1 | - | 1 | - |
| Decreased | 2.41 (1.67-3.47) | < 0.001 | 1.68 (1.21-2.32) | 0.002 | 1.56 (1.12-2.20) | 0.010 |
| **Cancer** |  |  |  |  |  |  |
| Normal | 1 | - | 1 | - | 1 | - |
| Decreased | 2.86 (1.51-5.42) | 0.002 | 2.19 (1.31-3.68) | 0.003 | 2.03 (1.14-3.62) | 0.016 |
| **CVD^a^** |  |  |  |  |  |  |
| Normal | 1 | - | 1 | - | 1 | - |
| Decreased | 2.92 (1.19-7.17) | 0.020 | 1.33 (0.56-3.13) | 0.516 | 1.35 (0.59-3.11) | 0.472 |

Note. All model were adjusted for age, sex, educational levels, family poverty to ratio, sleep duration, physical activity, smoking status, alcohol drinking, cardiovascular diseases and diabetes. ^a^ time-dependent binary GNRI was included in model.
