# Supplementary material for: The associations between the Geriatric Nutritional Risk Index and all-cause, cancer-specific, and cardiovascular mortality in the U.S. population: a large-scale pooled survey
Source: Nutr Metab (Lond). 2024 Jul 12;21:48. doi: 10.1186/s12986-024-00827-7 (PMC11245820; doi:10.1186/s12986-024-00827-7)
Supplement: Supplementary file 2 — Supplementary Material 2 [file 12986_2024_827_MOESM2_ESM.docx]

**Table S2.** The association of GNRI with all-cause mortality, cancer mortality, and cardiovascular diseases mortality using weighted cox regression after excluding the participants under 1-year visit.

| **Mortality** | **CKD** | |
| --- | --- | --- |
|  | **HR (95% CI)** | ***P* value** |
| **All-cause** |  |  |
| Normal | 1 | - |
| Decreased | 1.59 (1.17-2.17) | 0.004 |
| **Cancer** |  |  |
| Normal | 1 | - |
| Decreased | 2.01 (1.23-3.28) | 0.006 |
| **CVD^a^** |  |  |
| Normal | 1 | - |
| Decreased | 1.33 (0.82-2.13) | 0.242 |

Note. All model were adjusted for crude age, sex, educational levels, family poverty to ratio, sleep duration, physical activity, smoking status, alcohol drinking, cardiovascular diseases and diabetes. ^a^ time-dependent binary GNRI was included in model.
