# Supplementary figures and images for: The associations between the Geriatric Nutritional Risk Index and all-cause, cancer-specific, and cardiovascular mortality in the U.S. population: a large-scale pooled survey
Source: Nutr Metab (Lond). 2024 Jul 12;21:48. doi: 10.1186/s12986-024-00827-7 (PMC11245820; doi:10.1186/s12986-024-00827-7)

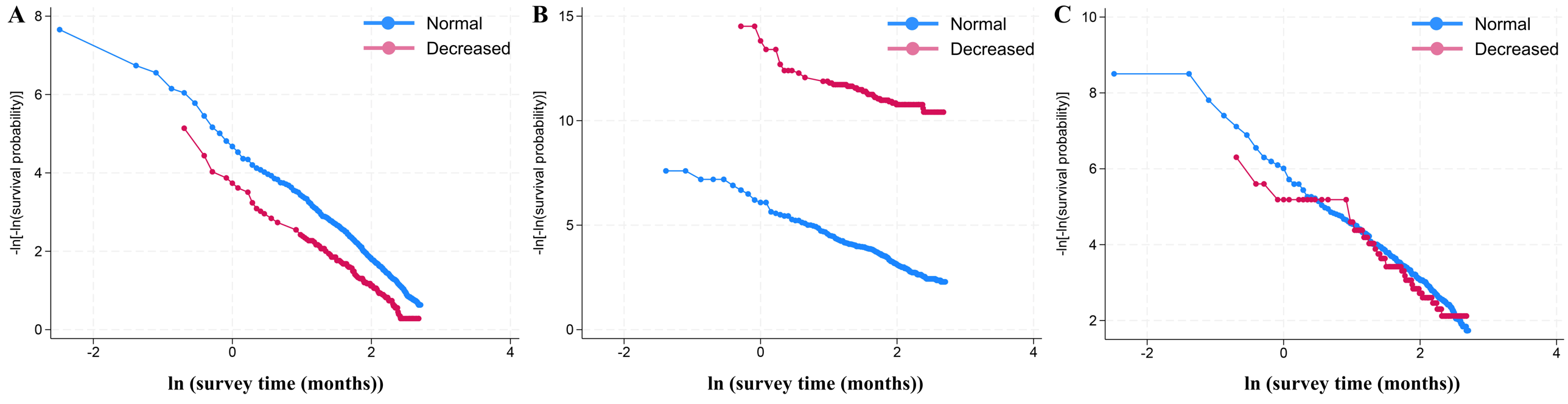

Supplement: Supplementary file 3 — Supplementary Material 3 [file 12986_2024_827_MOESM3_ESM.jpg]
